# Textile microfibers valorization by catalytic hydrothermal carbonization toward high-tech carbonaceous materials

**DOI:** 10.1016/j.isci.2024.111427

**Published:** 2024-11-19

**Authors:** Silvia Parrilla-Lahoz, Marielis C. Zambrano, Joel J. Pawlak, Richard A. Venditti, Tomas Ramirez Reina, Jose Antonio Odriozola, Melis S. Duyar

**Affiliations:** 1 School of Chemistry and Chemical Engineering, University of Surrey, GU2 7XH Guildford, UK; 2 Department of Forest Biomaterials, College of Natural Resources, North Carolina State University, Raleigh, NC 27695-8005, USA; 3 Inorganic Chemistry Department & Materials Science Institute, University of Seville-CSIC, Avda. Américo Vespucio 49, 41092 Sevilla, Spain

**Keywords:** Engineering, Materials science, Materials chemistry

## Abstract

Microplastics fibers shed from washing synthetic textiles are released directly into the waters and make up 35% of primary microplastics discharged to the aquatic environment. While filtration devices and regulations are in development, safe disposal methods remain absent. Herein, we investigate catalytic hydrothermal carbonization (HTC) as a means of integrating this waste (0.28 million tons of microfibers per year) into the circular economy by catalytic upcycling to carbon nanomaterials. Herein, we show that cotton and polyester can be converted to filamentous solid carbon nanostructures using a Fe-Ni catalyst during HTC. Results revealed the conversion of microfibers into amorphous and graphitic carbon structures, including carbon nanotubes from a cotton/polyethylene terephthalate (PET) mixture. HTC at 200°C and 22 bar pressure produced graphitic carbon in all samples, demonstrating that mixed microfiber wastes can be valorized to provide potentially valuable carbon structures by modifying reaction parameters and catalyst formulation.

## Highlights

• Microfibers can be upcycled toward carbon materials by catalytic HTC
• Fe-Ni catalyst can convert microfibers to filamentous solid carbon nanostructures
• Graphitic formation was detected in all samples at 200°C and 22 bar pressure

## Graphical abstract

**Figure:**
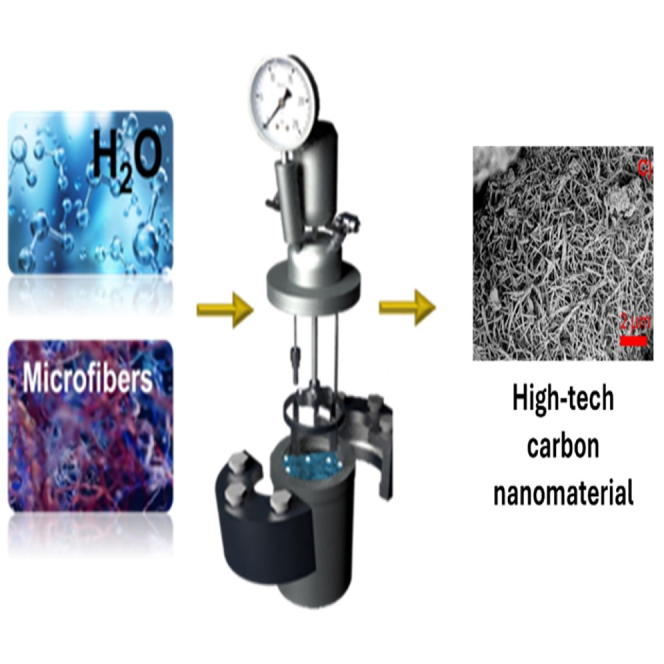

## Introduction

The consumption of plastic has expanded significantly and is anticipated to triple during the next two decades. Plastics production grew from 2 million tons in 1950 to 390.7 million tons in 2021.^1^ Plastics are prevalent in many aspects of our everyday lives, including textiles, cosmetics, transportation, and medicine. The global production of thermoplastics is expected to reach 445.25 million metric tons by 2025.^2^ 40% of the plastics produced are “single use” which means while they will become waste almost immediately, the waste biodegrades within 500–1000 years, remaining much longer in the environment.^3^^,^^4^ Microplastics are defined as plastics having a diameter of less than 5 mm and these can be produced either as a final product (primary microplastics) or through the deterioration of bulk plastics (secondary microplastics).^5^^,^^6^ In recent years, microplastics have attracted attention owing to their abundance and adverse impacts on the environment, which affect humans, animals, and ecosystems.^7^

Multiple studies reveal that textile microplastics (microfibers) make up 35% of primary microplastics released to the waters, which is a worsening problem due to the rise of fast fashion which relies on synthetic textiles.^8^^,^^9^ The two most prevalent fibers in the textile industry are polyethylene terephthalate (PET) and cotton; PET accounts for 52% of the worldwide market, with cotton following closely at 24%.^10^ Microfibers are released to the environment immediately when washing clothes, leading to an estimated 0.28 million tons of microfibers per year reaching aquatic environments.^11^^,^^12^^,^^13^ Therefore, several places around the world (France,^1^^,^^14^^,^^15^ Australia,^16^ and California^17^) are already implementing strategies to fight microfibers emissions by making mandatory the installation of microfibers filters in all commercial washing machines. While this is a positive step, there are currently no mitigation measures to ensure waste collected will not make its way back into the environment.

Recently, we have investigated the feasibility of valorizing this waste as a feedstock to generate valuable products using catalytic upcycling by pyrolysis.^18^ While most plastics waste upcycling research typically targets fuels, lubricants and chemicals as final products, due to the high surface area, filamentous microstructure, predictable composition and relatively clean nature of textile microfiber waste compared to other sources of plastic waste, we propose that catalytic upcycling to high-value/low-volume products such as carbon nanomaterials can be a feasible way to incorporate this waste into the circular economy. The increased surface area compared to other plastic wastes can improve reactivity, permit better heat and mass transfer, and enable better interaction with catalysts. In this work we use catalytic hydrothermal carbonization (HTC) of model microfibers from cotton and polyester (PET) at moderate temperatures and pressures to synthesize carbonaceous materials.

Modern HTC applications have employed different waste types of biomasses, municipal solid waste, plastics, and bulk textiles as the reactant, with the goal of producing solid carbon, various gases (including CO_2_, CO, CH_4_, and C_2_H_6_), and oil products.^19^^,^^20^ Studies have illustrated that incorporating PVC into the HTC process yields a range of compounds, including aliphatic and alicyclic hydrocarbons, benzene, naphthalene, diphenyl, phenanthrene, pyrene, and their alkyl derivatives. Additionally, valuable platform chemicals like acetic acid, furfural, lactic acid, propionic acid, phenolic compounds, hydroxymethylfurfural, levulinic acid, formic acid, and succinic acid can be recovered from the resulting hydrochar.^21^^,^^22^ During HTC, organic compounds undergo hydrolysis into low molecular weight molecules, which then re-polymerize into compounds with higher molecular weights due to their instability and reactivity. Furthermore, research has demonstrated that HTC conducted without a catalyst can effectively produce hydrochar. For instance, in a specific investigation, infant diapers were subjected to temperatures ranging from 200°C to 300°C for 3 h.^23^ In a different study, four types of plastics (polyethylene, PET, PP, and nylon) underwent HTC at moderate temperatures ranging from 200°C to 300°C, with a residence time of 3 h, employing seawater as the solvent.^24^ Furthermore, as the process temperature increased (200°C, 250°C, and 300°C), there was an observed increase in the carbon content of the hydrochar (from 77% to 80%), accompanied by a decrease in its oxygen content from 1.22% to 0.77%.^25^ Another study investigated HTC using both cotton and synthetic fibers in an autoclave reactor, where deionized water served as the solvent. The reactor temperatures ranged from 230°C to 280°C, with residence times of 30, 60, and 90 min. Findings from the study indicated a rise in carbon percentages by 10% under the operation condition of 280°C and 90 min, suggesting dehydrogenation and deoxygenation of the feedstock.^20^^,^^24^ Furthermore, co-hydrothermal carbonization has emerged as a potential technique for processing mixtures of different feedstocks, such as biomass and plastic.^26^^,^^27^ Co-processing biomass with synthetic polymers may help achieve a more balanced composition of carbon, oxygen, and hydrogen in the feedstock. This, in turn, would have a major impact on the characteristics of the resulting breakdown products.^28^ In the present study, it has been hypothesized that the presence of biomass (in this case, cotton) might enhance the radical degradation process by accelerating the decomposition of synthetic macromolecules. This is attributed to the comparatively poorer heat stability of biomass in comparison to polymers (in this case PET).^29^^,^^30^ In addition, the processes of hydrothermal and ionothermal carbonization (HTC and ITC) enable the conversion of low-cost biomass into functional carbon-based products at relatively low temperatures (180°C–220°C), making them efficient methods for producing carbon nanomaterials.^31^ To produce electrode materials for supercapacitors, a one-step ionothermal carbonization process that uses an iron-based ionic liquid as a solvent and a porogenic agent has been used.^32^ Table 1 summarizes all studies considered as comparing reaction temperature, residence time, and carbon output.

**Table 1:** Literature review summary

Feed	Temperature (°C)	Residence time	OUTCOME	Reference
Waste textile	230–280	30,60,90 min	Char (Increase carbon content 49–60 wt. %)	Lin et al.^20^
PVC waste	200, 230, 260	60 min	Char (Increase carbon content 37–82 wt. %)	Yao et al.^21^
Coal-biomass blend	200,230,260	30 min	Char (Increase carbon content 48–70 wt. %)	Saba et al.^27^
Disposable diapers	200–300	180 min	Char (Increase carbon content 60–84.5 wt. %)	Budyk and Fullana^23^
Marine plastic debris	200,250,300	180 min	Char (Increase carbon content 77–81 wt. %)	Iñiguez et al.^24^
Waste textile with waste wood	240	90 min	Char (Increase carbon content 38–63 wt. %)	Lin et al.^26^
Biomass-plastic (Almond plants with LDPE)	Max 360-500	90 min	Char (Increase carbon content 29–72 wt. %)	Adeniyi et al.^30^
D-fructose in FeCl_4_	180	6 h	Carbon particles (20–50 nm)	Lin et al.^32^
Jujun grass in FeCl_4_	180	10 h	Electrodes for super capacitors	Liu et al.^31^
PVC waste	180–260	15 h	Dechlorinated char	Poerschmann et al.^33^
HTC PET/cotton	200	12 h	Carbon nanomaterials (Graphite)	Current research

Herein, we use an Fe-Ni bimetallic catalyst to explore the feasibility of growing carbon nanomaterials under relatively mild HTC conditions from mixed microfiber waste containing PET and cotton. As mentioned in the introduction, PET and cotton were selected as the feedstock due to their widespread use in the textile industry. The aim is to examine the impact of HTC on each feedstock individually, as well as their combined effects when blended. Since the microfibers become too small to be sorted prior to the upcycling process and have been demonstrated by other authors that co-HTC may improve the quality of the carbon products, it is informative to test co-HTC as a realistic process for microfiber waste valorization. Regarding the catalyst selection, while Fe-Ni catalysts are used in a variety of catalytic processes, they have until now not been used for the co-HTC of textile-derived microfibers. The Fe-Ni catalyst is not a typical selection in the catalytic HTC of biomass literature, as most of the prior works have focused on synthesis of fuels using alkaline catalysts, such as carbonates and hydroxides of potassium, sodium, and calcium, organic acids such as acetic and formic acid, and inorganic acids such as sulfuric acid^34^*.* Transition metals like Ni, Fe, and Co efficiently generate H_2_ or CNTs from hydrocarbon feedstocks and solid waste by effectively breaking C–C and C–H bonds, at a lower cost than noble metals.^35^^,^^36^^,^^37^^,^^38^ By integrating a catalyst into the mild HTC reactor, we focus here on generating solid carbon products that can be used in applications such as separations, electronics, and sensors.

Even though the current research on HTC of PET/cotton blends requires a rather long residence time (12 h), the mild temperature of 200°C along with the use of a Fe-Ni catalyst offers substantial advantages to enhance the carbonization process and encourage the creation of well-structured graphite. When compared to previous investigations (Table 1), the fabrication of carbon nanomaterials—specifically, graphite—demonstrates comparable performance. However, chars from other research that use temperatures as high as 300°C and residence periods as short as 30 min sometimes turn out to be of lesser quality. For example, chars made from feedstocks such as used nappies or marine plastic waste may have a higher carbon content, but their structural integrity and potential uses are not as those of the nanomaterials produced.

## Results and discussion

**Figure 1:**
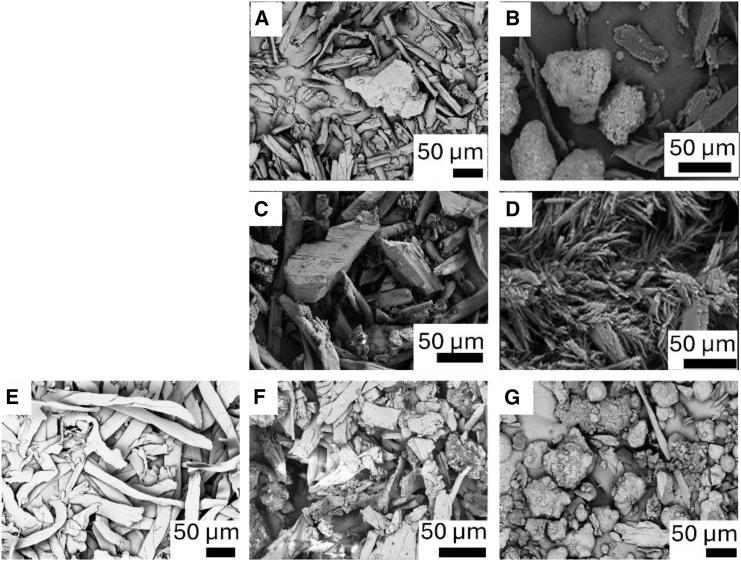
SEM image of all samples, including pre-reaction, post-non-catalytic reaction and post-catalytic reaction pre-reaction samples (E) PET/cotton physical mixture, post-non catalytic reaction samples of (A) cotton (C) PET (F) PET/cotton and post catalytic reaction samples of (B) cotton (D) PET (G) PET/cotton. Pre-reaction sample images for cotton and PET can be found in our previous publication, as the pre-reaction materials used for the study are the same.^18^ Particle size distribution Figure S5.

SEM was used to examine the surface morphology, microstructure of the microfibers and to characterize the growth/deposition of carbon nanostructures on the catalyst. Figure 1 shows SEM images of pre-reaction samples as well as products of catalytic and non-catalytic HTC. In Figure 1 for (A) Cotton, (C) PET and (F) cotton/PET are shown the products obtained after a non-catalytic HTC reaction. It can be observed that the SEM for post-non-catalytic HTC of PET (Figure 1D) no longer displays microfibers. In the case of the cotton, non-catalytic HTC results in the retention of a microfiber structure (Figure 1A), although the sizes of the microfibers appear smaller. The same structure is observed on the cotton/PET sample post-HTC (Figure 1G), which clearly resembles a mixture between products of PET and cotton HTC (Figure 1A and C). This indicates that the conditions employed in this study (200°C) are too mild to achieve full carbonization in the absence of a catalyst. In the case of catalytic HTC, the SEM images show the appearance of fibrous structures for PET after HTC (Figure 1D), which is distinct from that observed pre-reaction, while for cotton and PET/cotton mixture the post reaction sample displays a mixture of the microfibers and catalyst (spherical particles). In the case of cotton there are still some unreacted microfibers mixed with some carbonaceous products, which will be discussed in later sections in detail.

Table 2 provides an examination of pre-, post-non catalytic, and post-catalytic HTC carbon feed and products based on their oxidation temperatures. The catalytic post-reaction products of the PET sample resulted in a notable reduction in the amorphous phase and a rise in the filamentous phase relative to both pre-catalytic and non-catalytic conditions (from 15% to 71%). In contrast, the distribution of the cotton sample remained consistent at all stages due to the non-conversion achieve in the samples at these mild temperatures, this is clearly observed in the SEM images (Figures 1A–1C). After catalytic post-treatment, the PET/cotton sample exhibited a significant rise in the amorphous phase from 46% to 87% and a decrease in the filamentous phase from 54% to 13% compared to pre-catalytic and non-catalytic conditions.

**Table 2:** Total solid carbon content and carbon production calculated from TGA data

Total solid carbon production		
Sample	Amorphous (wt. %)	Filamentous (wt. %)
PET pre-	85	15
Cotton pre-	77	23
PET/cotton pre-	46	54
PET non-catalytic	66	34
Cotton non-catalytic	83	17
PET/cotton non-catalytic	55	45
PET catalytic	29	71
Cotton catalytic	85	15
PET/cotton catalytic	87	13

See Table S1 for details. The instrument specifications have a dynamic temperature precision of ±0.5°C, a weighting accuracy of ±0.5% and a weighting precision of ±0.1%. The error in the weight % calculations for the table from the TGA results is ±0.5%.

**Figure 2:**
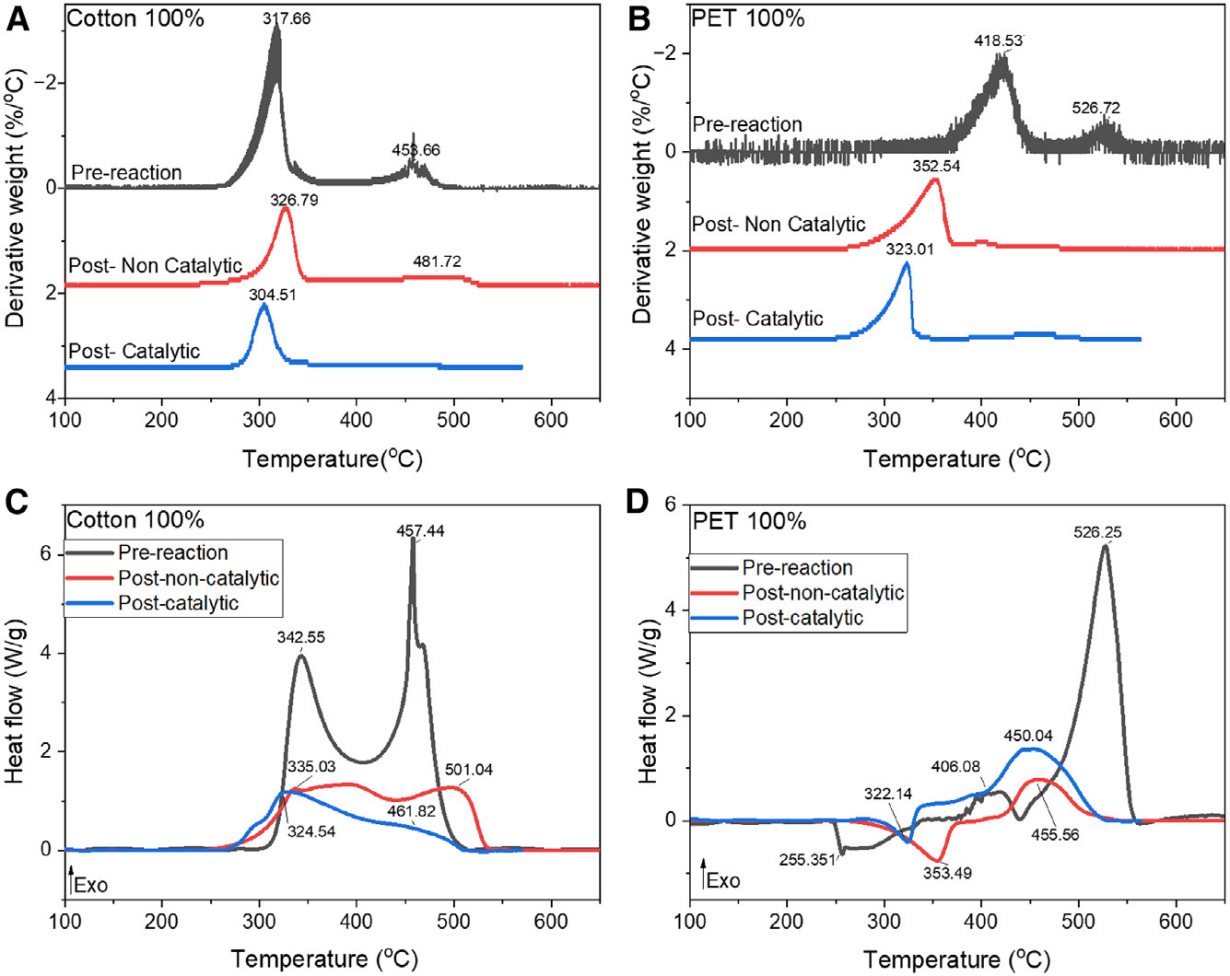
TGA-DSC curves of PET and cotton model samples (A and B) Derivative weight curves from TGA data of samples in air atmosphere (5°C min^−1^). (A) cotton (B) PET. (C and D) DSC curves of pre- and post-reaction samples in an air atmosphere (5°C min^−1^) (C) Cotton (D) PET. The instrument specifications have a dynamic temperature precision of ±0.5°C, a calorimetry accuracy/precision of ±2% (based on metal standards), a heat capacity accuracy ±5%, a weighting accuracy of ±0.5% and a weighting precision of ±0.1%.

Figure 2 shows the derivative weight plots (extracted from TGA data Figures S1 and S2) and differential scanning calorimetry (DSC) plots from temperature programmed oxidation (TPO) of as received textiles as well as HTC products. All weight lost before 100°C was attributed to water evaporation. The TPO of carbon products from non-catalytic and catalytic HTC experiments were analyzed by TGA-DSC to determine the quality and quantity of products of reaction (Figure 2). Generally, when polymers are subjected to TPO experiments, three main areas can be detected, the first one at low temperatures is a glass transition, where the polymer transitions from a hard and brittle state to a viscous state. After that the so-called melting processes are usually observed and finally the decomposition process takes place at higher temperatures depending on the polymer type.^39^ For our experimental data, the combustion of carbonaceous products is divided into three stages: first the evaporation of water and volatile mater, up to 100°C, second, the combustion of organic matter and carbohydrates, and lastly the combustion of carbonaceous matter (Data in Table S1).^40^ As the degree of carbonization increases, an increase of the ignition and combustion temperature of the carbonaceous products is observed. This happens due to the transition of the carbon structure from aliphatic to aromatic coalification after the HTC process.^40^ Co-hydrothermal carbonization (Co-HTC) is defined as when the HTC process feed is composed by biomass and plastic simultaneously. When co-HTC occurs, it has been reported that there is a shift toward higher combustion temperatures of the carbonaceous products.^40^

The DSC of cotton pre-reaction (Figure 2C) shows a first peak between 300°C and 400°C reaching a maximum at around 342°C. This exothermic reaction peak is attributed to the combustion of cellulose.^41^ This peak indicates a phase transformation due to the destruction of the crystalline part of the cellulose. The intensive thermal combustion of the cotton fiber is characterized by the major weight loss percentage observed in the derivative weight plot (Figure 2A pre-reaction curve), the total solid carbon weight loss for this event is 77% (Table 2). This first peak characterizes the surface ignition of the material. The main component of cotton is cellulose, which is a natural polymer with a carbon content of 44 wt. %. During TPO the destruction of cellulose occurs in different stages. Between 200°C and 230°C the amorphous component mostly disintegrates while the crystalline component remains. The degradation process begins when the temperature reaches 270°C–280°C or above.^41^ A second peak is detected in the DSC plot of cotton pre-reaction sample (Figure 2D) reaching a maximum at 460°C, attributed to the re-ignition of the material, corresponding to a weight loss of total solid carbon of 23%. During TPO of cotton, at about 340°C, full amorphization takes place, accompanied by a maximum weight reduction of around 60%. The coal structure is then produced during the TPO process as the transformation from amorphous to carbonized cellulose structure begins.^41^ The DSC curves of cotton post-reaction of catalytic and non-catalytic experiments (Figure 2D) show two different exothermic processes. For the case of the non-catalytic post-reaction carbonaceous products, there are two low temperature exothermic reactions at 335°C and 400°C, with a weight loss observed in Figure S2 of total solid carbon of 66%. The third peak reached its maximum at 500°C. Mass loss of total solid carbon detected between 400°C and 500°C was 34% (Figure S2).

In the case of the catalytic reaction of cotton, (blue line, Figure 2D ) the initial exothermic peaks were detected around 300°C with a mass loss of total solid carbon of 76% and a smaller exothermic peak was detected at 460°C. In comparison with the pre-reaction cotton sample, there is an increase of the combustion temperature for the non-catalytic reaction products and a slightly lower temperature of combustion for the post-catalytic HTC sample. Summarizing the previous information, the experimental data obtained for cotton shows that carbonization can be observed for the non-catalytic HTC products.

Figure 2B and 2D show the derivative weight and DSC plots for PET pre-reaction microfiber and post-catalytic and non-catalytic products. The DSC plot for the pre-reaction PET microfibers (Figure 2D, Blue line) shows an initial endothermic event due to the PET melting (no weight change) at 255°C followed by several exothermic peaks attributed to the combustion of polyester. The first exothermic peak is positioned at 400°C accounting for a total mass loss of solid carbon of 85%. The second exothermic peak due to oxidation reactions of char, is positioned at 526°C with a mass loss of total solid carbon of 15 wt. %.^42^

The DSC curves of PET post-reaction (Figure 2D) show two different processes, an initial endothermic weight loss event followed by a second exothermic weight loss event at higher temperatures. For the case of the non-catalytic post-reaction carbonaceous products an endothermic event at 353°C is shown with a weight loss of total solid carbon of 78%. A second exothermic event is detected with it peak at 455°C with a weight loss of 22%. For the catalytic post-reaction sample the same reactions were detected at slightly lower temperatures, 320°C and 450°C, respectively. The sharp endothermic peak observed in both post-reaction samples is attributed to an evaporation or thermal decomposition of organic matter trapped in the material during HTC.^43^ This is supported by the weight loss observed in the derivative weight plot. Other authors have reported that activated carbon has a desorption peak around 300°C.^44^ Hence the PET derived products after HTC at 200°C and 12 h can be activated carbon or other high surface area carbonaceous components with adsorption properties. It has been reported in the literature that weight loss due to exothermic events occurring between 400°C and 700°C is attributed to the oxidation of the carbon skeleton of graphene sheets and over 500°C can be a mix between graphite and graphene.^45^ In addition, according to relevant literature, the weight loss observed after 450°C can be attributed to graphitic carbon with strong thermal stability.^46^

**Figure 3:**
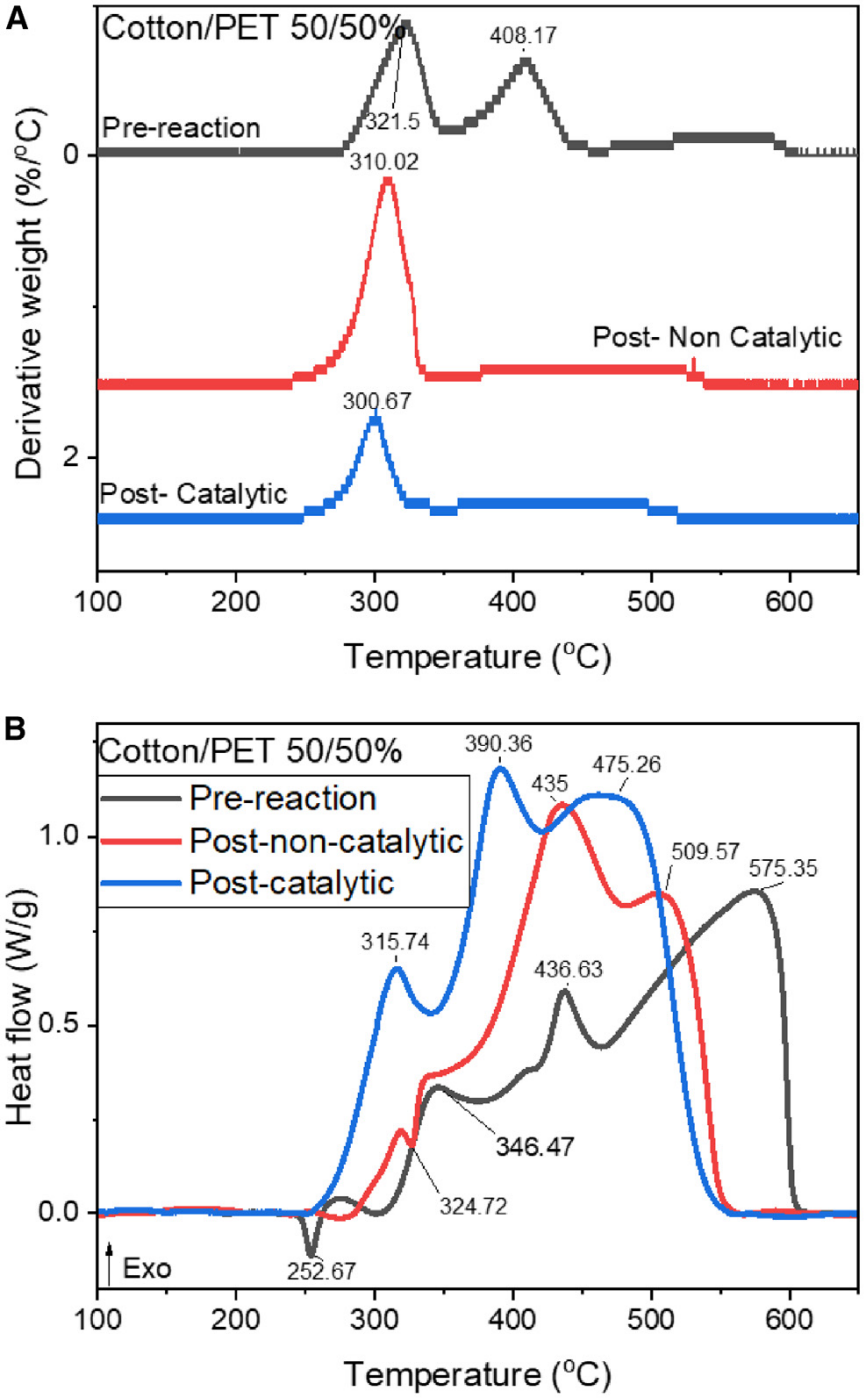
TGA-DSC curves of 50%/50% PET/cotton model sample (A) Derivative weight curves of blend PET/cotton from TGA data of samples in air atmosphere (5°C min^−1^). (B) DSC curves for blend PET/cotton of pre- and post-reaction samples in air atmosphere (5°C min^−1^). The instrument specifications have a dynamic temperature precision of ±0.5°C, a calorimetry Accuracy/precision of ±2% (based on metal standards), a heat capacity accuracy ±5%, a weighting accuracy of ±0.5% and a weighting precision of ±0.1%.

For experiments involving the cotton/PET blend, the derivative weight plot and DSC plot are shown in Figure 3A and 3B. In the case of the pre-reaction microfibers there is a superposition of peaks related to PET and cotton. The decomposition of cellulose is expected to be shown by the loss mass starting before 300°C and ending approximately around 350°C and the combustion of PET is expected to be observed between 350°C and 450°C.^42^ The initial peak for the pre-reacted microfibers on the DSC plot (Figure 3B black line) is attributable to the melting of PET at 252.7°C. The primary process involved in cotton oxidation is denoted by the location of the exothermic peak reaching a maximum at 341°C. This peak has a weight loss of solid carbon of 46% (Table 1). Moreover, the exotherm associated with PET oxidation is detected at 436°C, with a weight loss of solid carbon of 54%. A broader peak was detected at 575°C that is attached to the re-ignition of the cellulose and the oxidation reactions of char.^42^

**Figure 4:**
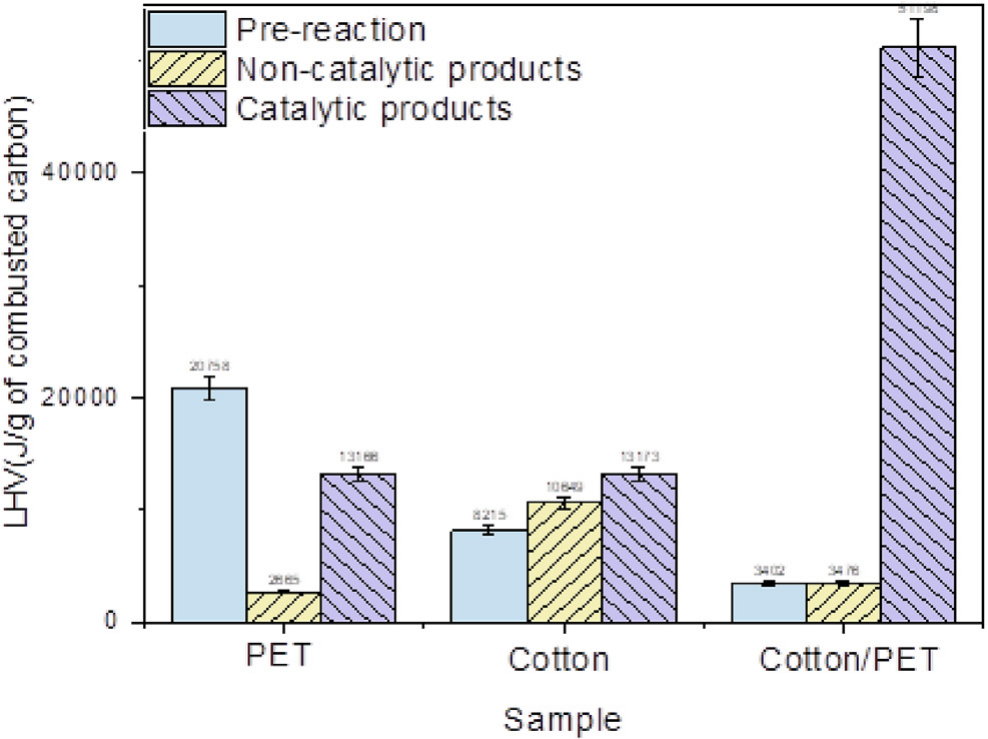
Comparative plot of heating values of all samples from pre-reaction in light blue to non/catalytic post-reaction products in yellow to post-catalytic normalized post-reaction products in purple Data obtained from Table S2. (Data are represented as mean of ±5%).

The DSC curves of the cotton/PET blend post-reaction products (Figure 3B) show three different thermal events. For the non-catalytic reaction products, an endothermic peak is detected at 324°C due to the evaporation/decomposition of organic matter adsorbed/retained by the sample; the weight loss of desorbed organics is of 24 wt. % (Table S1). As it has been reported before, relevant literature reported that PET can be utilized as feed to produce activated carbon by carbonization, therefore the low temperature peaks observed int the TGA plot and endothermic reaction observed around 300°C can be attributed to the desorption of organic volatile matter stored in the activated carbon products.^39^^,^^46^^,^^47^ Two exothermic events are detected at higher temperatures, the first at 435°C and the second one at 509°C. The weight loss of total solid carbon (Table 1) in this range is 55 wt. %. For the post-catalytic HTC sample, three exothermic peaks were detected. The first one at 315°C, due to the combustion of amorphous carbon synthesized with a weight loss attached of 50 wt. %. Two peaks were detected at higher temperatures, one at 390°C and the other one at 475°C. The increase in the LHV for the carbonaceous products of the cotton sample and the PET/cotton sample after the reaction in comparison to the pre-reaction sample (Figure 4) indicates good carbonization of the sample. As the present work is a proof of concept of HTC of microfibers, it is necessary to optimize the reaction conditions toward smaller residence times and at the same time the optimization of the design of the catalyst to improve performance. Figure 4 shows a comparative between heating values normalized by gram of combusted carbon.

**Figure 5:**
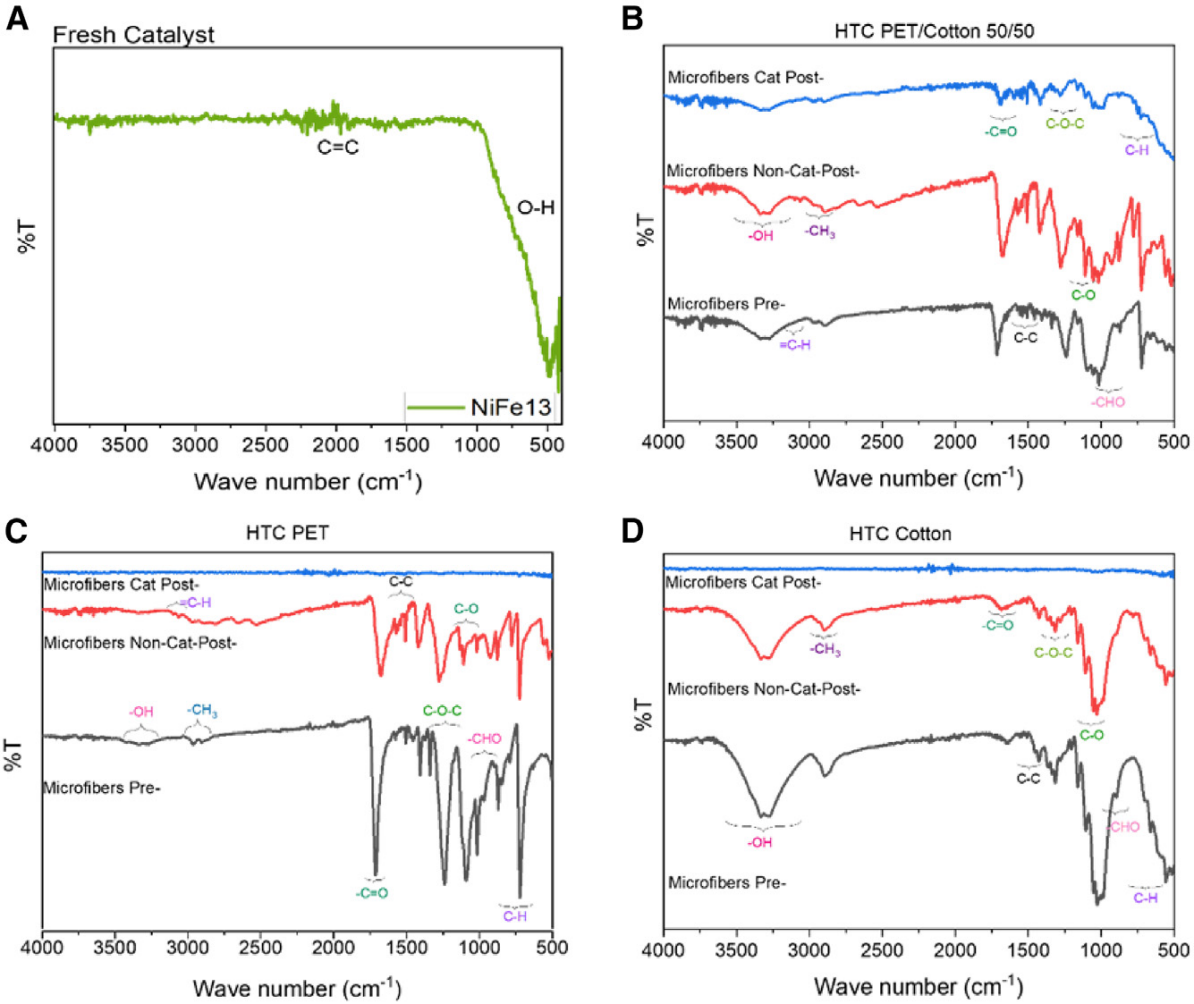
FT-IR (Fourier transform infrared) spectra curves of all samples, pre-post reaction samples For (A) fresh catalyst (B) PET/Cotton (C) PET and (D) cotton.

FTIR analysis was executed to characterize the alterations of functional groups upon performing HTC on the microfibers (Figure 5). This analytical approach can provide a detailed understanding of the changes occurring in the molecular structure, shedding light on the specific functional groups that experienced modifications during the process. It can be seen from Figure 5 that the samples after undergoing catalytic HTC present fewer or no functional groups associated with the original microfibers. This can be due to destruction of the microfibers and/or a dilution effect in the presence of the catalyst in this specific sample. It should be noted that a comparison of the PET/cotton mixture with PET only and cotton only samples post catalytic reaction, (Figures 5B–5D respectively), the PET/cotton mixture does display functional groups present in the original sample after catalytic HTC, whereas the pure PET and pure cotton cases do not display these peaks post catalytic reaction, indicating microfiber destruction contributes to the effect (rather than simple dilution alone). In Figures 5B–5D, it can be appreciated that in the non-catalytic experiments there is not a destruction of the initial microfibers indicating that the total conversion of the feed hasn’t been achieved, consistent with the previously discussed TGA and SEM results. The band detected around 2900–2970 cm^−1^ in Figures 5B–5D, is ascribed to the aliphatic –CH_3_ asymmetric stretching vibration. The intensity of the band decreases from the pre-reaction samples to the post-reaction non-catalytic sample, indicating a loss of –CH_3_ functional groups.

As shown in Figures 5B–5D, the wide bands at a wavenumber around 3200-3600 cm^−1^ are attributed to OH-stretching vibration bands of hydroxyl and carboxyl groups. This peak is not present in Figure 5C, for PET samples as if we look at the chemical structure of the PET, -OH functional groups are not characteristics. For PET pre-reaction sample (Figure 5C), the typical PET peaks are shown; 1730 cm^−1^ attributed to C=O stretching of carboxylic acid groups, at 1453 cm^−1^ and 1342 cm^−1^ indicating the C–H bending of ethylene glycol segment, at 1240 cm^−1^ shows the C–O stretching of carboxylic acid and at 712 cm^−1^ attached to the interaction of the benzene rings.^48^ The FT-IR spectrum of PET structural conformation have been studied by several researchers; it has been reported that an increase in the intensity of the 845cm^−1^, 973cm^−1^, 1,337cm^−1^, and 1,470 cm^−1^ peaks during crystallization is attributed to the *trans*-conformation of the ethylene glycol segment. On the other hand, a decrease in the intensity of the 895 cm^−1^, 1,040 cm-1, and 1,453cm^−1^ peaks is due to a gauche-conformation of the ethylene glycol segment. In addition, at 898cm^−1^ peak is representative of the amount of amorphous content of the PET polymer, due to the wagging of the oxy-ethylene group and their gauche and *trans* conformation. Moreover, peaks between 1,240 and 1,330 cm^−1^ are associated with parallel dichroism and peaks at around 1,729 cm^−1^ are associated with perpendicular dichroism.^49^ The absorption bands at 1,340 cm^−1^ and 1,370 cm^−1^ correspond to the wagging of the ethylene units in trans and gauche conformations, an increase in the intensity of the 1,340 cm^−1^ band also confirmed the assignment to the *trans* isomer.^49^ In general, the OH-vibrational band peak became less intense for the non-catalytic and catalytic HTC experiments than from the one recorded for the pre-reaction microfibers. Several researchers have shown that the decrease in the intensity of this peak can be due to dehydration and decarboxylation reactions in HTC reactions.^50^ Moreover, this indicates the decrease of hydroxyl and carboxyl contents contained in the microfibers yielding to H_2_O and CO_2_ improving the hydrophobicity of the carbonaceous products.^20^^,^^50^ For the aromatic groups observed, the absorbance of the bands at 3000–3100 cm^−1^ attached to the vibration of the aromatic benzene ring = C–H stretching vibration, appeared slightly in Figure 5B and 5C for the non-catalytic post-reaction products and to a lesser extent for the catalytic post-reaction products. The band between 1460 and 1600 cm^−1^ is attributed to the aromatic C–C stretching vibrations and has the higher intensity for all the samples in the non-catalytic post-reaction products, indicating a higher degree of aromatization for the carbonaceous products in this specific case. It is observed with higher intensity for the physical mixture of PET/cotton (Figure 5B) revealing the occurrence of aromatic reactions during the HTC process. The band located at 1100–1160 cm^−1^ is attributed to aliphatic ether C–O or alcohol C–O stretching vibration. The decomposition of these groups indicates the occurrence of decarboxylation reactions and production of CO and CO_2_ would be expected during the HTC reaction of textiles microfibers. The peak between 900 and 980 cm^−1^ is attributed to aldehyde -CHO stretching vibration.^20^^,^^48^^,^^50^

**Figure 6:**
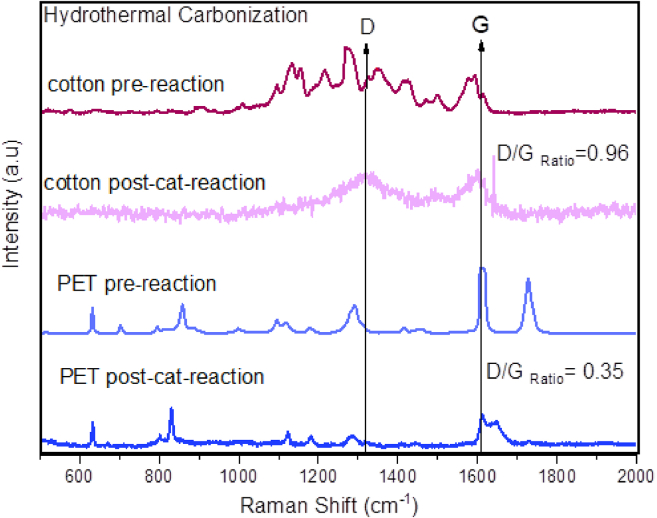
Raman spectra for cotton and PET pre-reaction microfibers and post-catalytic reaction carbonaceous products

Raman spectroscopy was employed to analyze the molecular structure, chemical bonding, and composition of both textile fibers (cotton and PET) and HTC post-catalytic reaction products, providing insights on the types of carbon formed on the catalyst. Figure 6 presents Raman spectra obtained from textile fibers and HTC products. In the Raman spectrum of PET pre-reaction microfibers, two dominant bands were detected, the first one at 1,600 cm^−1^ attributed to the presence of C–C aromatic rings and the second one at 1,725cm^−1^ attributed to the carbonyl C=O bond stretching. There are additional bands, one at 702cm^−1^ is due to ring C–C stretch, and another one detected at 859cm^−1^ is due to the vibration of C–C bonds and COC bending. The band at 1,096cm^−1^ represents the C–O and COC stretching, at 1181 cm^−1^ is the C–C ring stretching and at 1,289 cm^−1^ is identified the CO–O stretch.^51^ An additional band (around 600 cm^−1^) has been observed for both pre-reaction and post-catalytic HTC sample of PET, due to the olefinic C–H bending and C=C twisting.^51^ If we compared the pre-reaction PET sample with the post-catalytic HTC product, particularly, the absence of the 702 cm^−1^,859 cm^−1^, 1,096 cm^−1^, 1,289 cm^−1^, and 1,725 cm^−1^ attributed to the vibrational mode of C–C ring stretch, C–C bonds, COC bending, C–O and COC stretch, CO–O stretch and the carbonyl C=O stretching, respectively, are indicative of deoxygenation and depolymerization reactions. While the peak at 1,600 cm^−1^ appears sharper indicating higher level of crystallinity and a new peak at 1,644 cm^−1^ is detected. The precise determination of the ordered and disordered structures of amorphous carbon materials is possible by Raman spectroscopy by means of an examination of the D and G features, which are characteristic of carbonaceous materials. Specifically, for carbons with high hydrogen content, the D and G peaks, are situated at around 1,300 and 1,600 cm^−1^. The vibrational mode of sp^2^-hybridized carbon atoms in graphene or graphite-like materials is often associated with the G band. The location of the G band, which corresponds to the in-plane vibrations of carbon atoms in a hexagonal lattice, might provide insights into the graphitic characteristics of carbon-based materials. Various factors may influence the precise location of the G band, including the quantity of graphene layers, the extent of graphitization, and the existence of defects or functional groups. Therefore, the appearance of this peak indicates carbonization (graphitization) during the HTC catalytic reactions of PET.^52^

**Figure 7:**
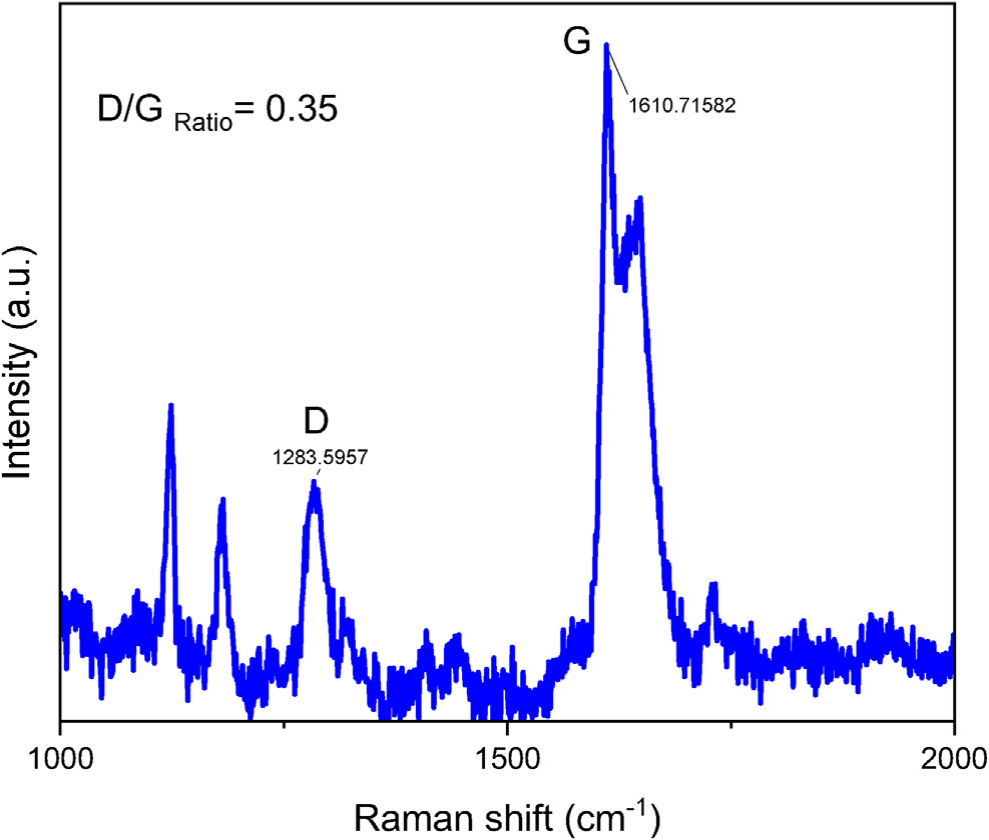
Raman spectra zoom-in for post-catalytic reaction PET carbonaceous products

**Figure 8:**
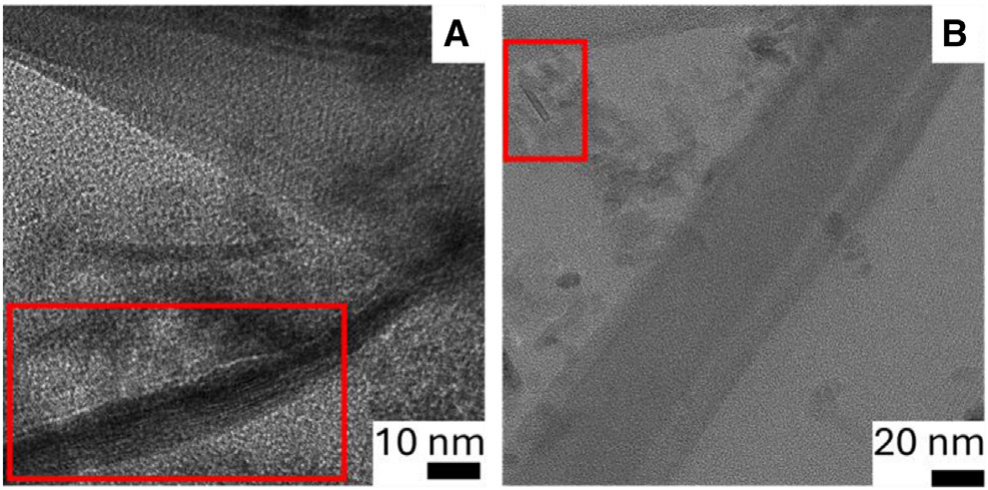
TEM images of cotton and PET post-reaction samples (A) cotton post-reaction catalytic sample (B) PET post-reaction catalytic sample. Layers of graphite indicated by the red squares.

**Figure 9:**
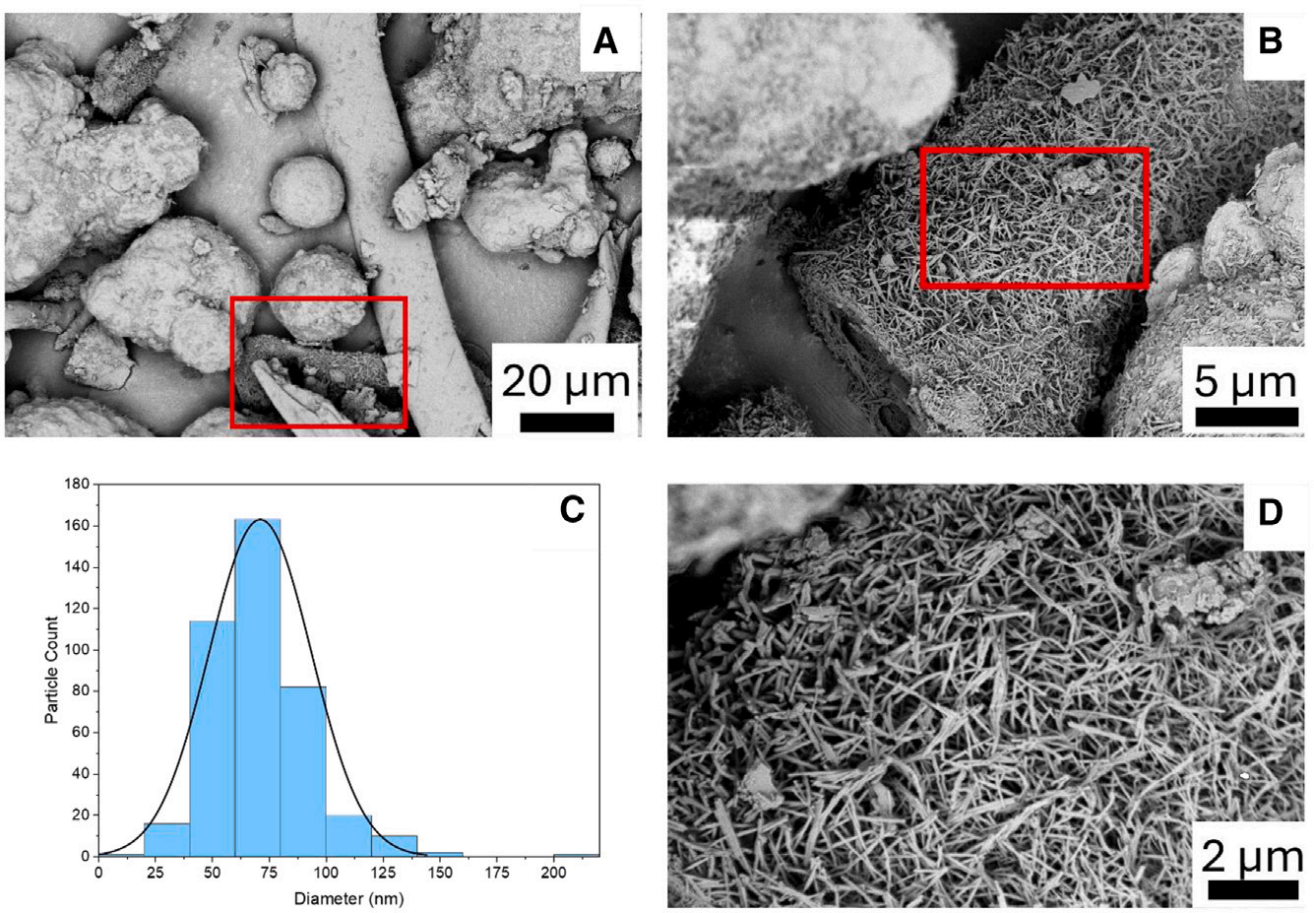
Carbon nanotube structures formed from PET/cotton after catalytic HTC reaction at 200°C and 12 h of residence time at different magnifications (A–C) (A) 20μm resolution image where delimited possible CNTs clusters, (B) 5 μm magnification of the red delimited area from (A) and (D) 2 μm magnification of delimited area from (B) with the diameter distribution at (C) (Data are represented as mean of ± 2.35).

In Figure 6 it can be appreciated the Raman spectra of the cotton and PET pre-reaction microfibers showing the typical Raman peaks for cotton fibers and in the post-reaction sample can be clearly appreciated the appearance of the characteristic D and G peaks of carbonaceous materials. The peak at 1,598 cm^−1^ for cotton products (Figure 6) and 1,610 cm^−1^ for PET products (Figure 7) (G band) is associated with the vibration of sp^2^-bonded carbon atoms inside a graphite layer and corresponds to an E_2g_ mode of hexagonal graphite. The D band seen at around 1,315 cm^−1^ for cotton products (Figure 6) and 1,283 cm^−1^ for PET products (Figure 7) is attributed to the vibrational motion of carbon atoms that possess dangling bonds near the plane terminations of disordered graphite.^45^ Defect concentration in the graphene plane is typically associated with the intensity of the ratio G/D band. The quantification of defects density in sp^2^ carbon atoms was achieved by calculating the intensity ratio of the D-band and G-band. For the post-catalytic reaction carbonaceous products from cotton, the D/G ratio is 0.96, this ratio has been reported in the literature as characteristic of graphene oxide.^53^ For the post-catalytic reaction carbonaceous products from PET, the D/G ratio is 0.35, this value suggests high level of graphitization in the material, in fair agreement with the SEM study (Figure 1F). Figure 8 shows transmission electron microscopy (TEM) images of (A) cotton post-reaction catalytic sample, (B) PET post-reaction catalytic sample, where it can be appreciated several layers of graphite (indicated by the red squares). Furthermore, Figure 9 shows possible carbon nanotubes synthesis for the products of the catalytic HTC reaction of PET/cotton at 200°C and 12 h.

**Figure 10:**
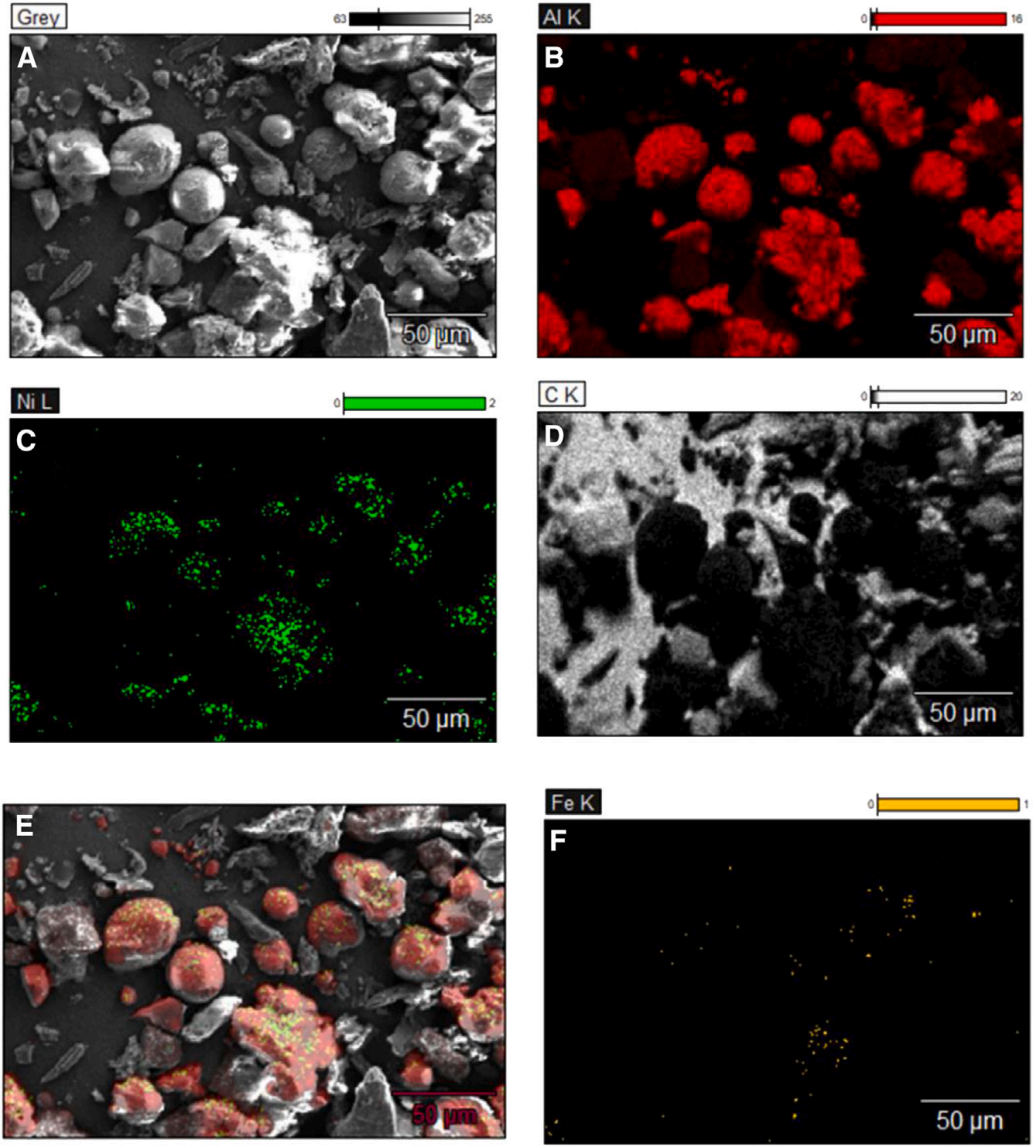
SEM-EDX images of PET/cotton post-reaction sample (A–F) (A) SEM image, (B) red indicates alumina (C) green indicates Ni, (D) indicates carbon, (E) is a combination of B, C, and F and (F) orange indicates iron.

SEM-EDX (Figure 10) was used to characterize the growth/deposition of carbon nanostructures on the catalyst. Carbon tape was used to fix the samples to the sample holder, therefore not all the carbon in image D is due to the carbon deposition from HTC. It can be appreciated that the sample surface is Ni-rich, as expected from the 3:1 Ni:Fe atomic ratio used during catalyst synthesis. The large catalyst particles and abundant Ni only regions (rather than Ni-Fe alloy) are detrimental for producing high value products such as carbon nanotubes. For future research stabilizing the particle size, catalyst dispersion and securing a Ni-Fe close contact are worth studying to optimize the yield and selectivity of desired carbonaceous products.

When comparing cotton and PET as materials for synthesizing carbonaceous materials, it is clear that PET is a superior precursor because of its clean composition with minimal impurities, whereas cotton contains lignin, cellulose, hemicellulose, and other organic components that can introduce impurities into the process. Based on the previous statement, it can be concluded that PET is a consistent carbon source that enables the production of carbon products with greater reproducibility and improved thermal stability. This allows for the synthesis of carbon products through carbonization while maintaining the structural integrity and purity of the carbon material. When PET is combined with cotton it enhances the carbon yield, and the cotton fibers with a porous structure can serve as a template for the creation of more structured or crystalline products like CNTs. The carbon percentage present on the microfiber can affect the quality of the products; as observed from the TGA results (Table 2), HTC of cotton led to a more amorphous product while HTC of PET to a more filamentous product. Table S5 displays the specific surface area of the samples before and after the reaction. While products of PET and cotton are thought to both have a significant contribution of surface area from the catalyst, it can be seen that the cotton-derived products possess a slightly higher surface area compared to those formed from PET. Furthermore, PET (pre-reaction) has a higher thermal stability in air compared to cotton (Figure 2C and 2D), which can also influence product formation during HTC. However, it should be noted that such adjustments will likely not be possible due to the mixed presence of the fibers. In this research paper, we provide a proof of concept for the synthesis of carbon products that can be utilized while potentially avoiding the CO_2_ emissions that would arise if these carbon products were to be used as solid fuel. This study showcases the synthesis of several carbon products, including highly porous amorphous carbon with notable adsorption capabilities, as well as graphitic carbon and carbon nanotubes. In the future, our goal is to decrease the production of amorphous carbon and optimize the technology to customize the manufacturing of graphitic carbonaceous products. Enhancements are required for the catalyst material to optimize the technology, including a focus on improving catalytic performance to maximize the production of CNTs or other graphitic carbon materials. In addition, the process reaction conditions should be optimized as well by increasing the temperature the degree of carbonization will be expected to increase, improving the retention of carbon as a solid state.

### Environmental and practical implications

The United Nations (UN) has introduced the Sustainable Development Goals (SDGs) as a comprehensive plan to attain a more prosperous and enduring future for everyone (UN). The SDGs are a collection of 17 worldwide objectives that were established by the UN in 2015 as a component of the 2030 Agenda for Sustainable Development. Among these 17 SDGs, co-HTC of textile microfibers contributes to SDG3 (good health and wellbeing), SDG6 (clean water and sanitation), SDG7 (affordable and clean energy), SDG9 (industry, innovation, and infrastructure), SDG11 (sustainable cities and communities), SDG12 (responsible consumption and production), SDG13 (climate action), SDG14 (life below water), and SDG15 (life on land). Valorization of textile microfibers waste can provide energy or value-added materials, thus incorporating this waste source into the circular economy.^54^

During 2018, the overall quantity of textiles and clothes discarded by the average American was roughly 47 kg. Out of the entire amount, only 15% underwent recycling or repurposing, while the remaining 85% was either disposed of in landfills or burnt.^55^ At present, microfibers face similar circumstances. Recycling provides a means to redirect old clothing and textiles away from landfills. Dry and clean materials, regardless of their poor condition, can be offered on secondary markets for utilization as rags or industrial fillers.^56^ Textile materials can be classified into four main techniques for recycling and recovery. Primary recycling is the process of recovering resources to create new goods that have the same value as the original ones. Secondary recycling involves using mechanical processes to recover elements from textile waste that have different physicochemical qualities. Tertiary recycling uses thermochemical techniques, such as pyrolysis, to transform textile waste into fuel. Quaternary recycling refers to the act of choosing an energy recovery conversion procedure.^57^ The recycling techniques mentioned before are ineffective in managing microfiber waste due to the need for sorting and the presence of pollutants like silica from laundry detergents.

Pyrolysis is a thermodynamic process that occurs in the absence of oxygen or air and requires an input of energy, is an endothermic reaction that occur a high temperature, resulting in a very energy intensive process.^57^ HTC, being an exothermic reaction happening at moderate temperature and autogenerated pressure, is less energy intensive. Furthermore, with other thermochemical technologies like pyrolysis prior drying treatment is necessary, on the contrary, the HTC method utilized herein allows for the processing of wet feed without the need for prior drying reducing operating costs.^58^

When considering scaling up of HTC technologies, process water management must be a significant consideration. The primary attribute of the process water is its high acidity, with a pH level ranging from 2.7 to 4.5.^59^^,^^60^ In designing industrial processes, considerations must be made for recycling the process water within the HTC process rather than generating large volumes of wastewater. In addition to minimizing the use of fresh water, this approach can enhance the yield of carbonaceous solid products, optimize energy efficiency, and enhance the environmental sustainability of the HTC process.^61^^,^^62^ Reforming the HTC process water to produce hydrogen and clean water is another approach that has been explored to treat wastewater.^63^^,^^64^ In addition to the liquid phase products, HTC also produces some CO_2_ gas which contributes to its carbon footprint.^65^

Several companies like Ingelia, AVA-CO2, CarboRem, SunCoal, Antaco, Carbon Solution, TerrNova, HTCycle, and C-Green have successfully developed industrial-scale plants with capacities ranging between 5000 T/year and 150000 T/year to upcycle biomass into solid fuel or chemicals,^66^ demonstrating the scalability of the process.^66^ In comparison, textile microfiber waste is estimated to be released at a rate of 200,000–500,000 tons/year worldwide from clothes washing activities.^67^

HTC upcycling of microfibers can be an economically viable and sustainable waste management approach due to the production of valuable by-products. Industries may use these variables to employ HTC technology to make a significant contribution to a circular economy. In order to fully harness the potential of this revolutionary waste management solution, it is imperative to invest in future research and development in HTC technology, as well as maintain ongoing governmental support.

### Conclusion

We have explored low temperature HTC as a means of addressing microplastics pollution from clothes washing. We demonstrated that microfibers derived from the two most often used textiles, cotton (natural fiber) and polyester (synthetic/polymer fiber), can be converted to solid carbon nanostructures using an Fe-Ni catalyst, and that low temperature HTC remains too mild to achieve destruction of the original structures in the absence of a catalyst. Several HTC reactions were performed at 200°C (22 bar) and 12 h of residence time for model microfibers of cotton, PET and mix 50/50 of cotton/PET. The solid products were analyzed using TGA-DSC, Raman, FT-IR, and SEM techniques, which revealed the conversion of the microfibers into a mixture of amorphous and graphitic carbon structures, including some carbon nanotubes formation from a mixture of cotton/PET. Graphitic carbon formation was detected in all samples following HTC at 200°C and 22 bar pressure, indicating that mixed microfiber wastes can be valorized to yield potentially useful carbon structures, by tuning reaction conditions and catalyst formulation.

### Limitations of the study

This study has several limitations related to the experimental methods employed. First, the reaction conditions in the laboratory-scale experiments may not accurately reflect those in industrial environments, potentially limiting the scalability and broader applicability of the findings. Furthermore, model samples of microfibers were used to simplify the study analysis, and certain factors, like impurities in the feedstock, were not considered, which could lead to discrepancies when applied in real-world scenarios. To improve the relevance of these results in industrial settings, future studies should explore pilot-scale experiments and incorporate variations in feedstock, such as actual microfibers waste.

## Resource availability

### Lead contact

Requests for further information and resources should be directed to and will be fulfilled by the lead contact, Dr Melis S. Duyar at m.duyar@surrey.ac.uk.

### Materials availability

This study did not generate new unique reagents.

### Data and code availability

Experimental data have been deposited at the University of Surrey open research repository and are publicly available as of the date of publication. Accession numbers are listed in the key resources table. This paper does not report any original code. Any additional information required to reanalyze the data reported in this paper is available from the lead contact upon request.

## Acknowledgments

Financial support for this work was provided by the University Global Partnership Network (UGPN) Research Collaboration Fund (RCF), the School of Chemistry and Chemical Engineering at University of Surrey and the Department of Material Science at University of Seville. University of Seville acknowledges financial support from the Spanish Ministry of Science through the projects NICER-BIOFUELS (ref: PLEC2021-008086), sponsored by MCIN/AEI/10.13039/501100011033 Next Generation Europe and SMART-FTS (ref: PID2021-126876OB-I00). The authors would like to acknowledge the help of the School of Chemistry and Chemical Engineering laboratory technicians Ben Gibbons, Thomas Chamberlain, and Yusuf El-Hassan for their continued support in the lab activities of S.P.-L. S.P.-L. also thanks Andreas Iakovidis of Mechanical Engineering Sciences, and Dr Rachida Bance-Soualhi of the department of Chemistry and Chemical Engineering for their assistance with SEM, and Raman, respectively. S.P.-L. acknowledges the University of Surrey Breaking Barriers Fellowship for funding her doctoral studies.

## Author contributions

S.P.-L.: writing the original draft, conceptualization, visualization, and investigation. M.C.Z.: investigation. J.J.P.: funding acquisition, supervision. R.A.V.: funding acquisition, supervision. J.A.O.: funding acquisition, conceptualization, supervision. T.R.R.: funding acquisition, conceptualization, project administration, writing e review and editing and supervision. M.S.D.: funding acquisition, supervision, conceptualization, visualization, project administration, and writing e review and editing.

## Declaration of interests

The authors assert that they do not have any conflicting interests. S.P.-L., M.S.D., T.R.R., J.J.P., and R.A.V. are identified as inventors on an international patent application (Patent number: PCT/GB2023/051538) associated with certain aspects of the research discussed in this publication. The patent application is owned by the University of Surrey and North Carolina State University. The authors state that the presence of this patent does not impact the analysis or communication of the study results in this work.

## STAR★Methods

### Key resources table

**Table:** 

REAGENT or RESOURCE	SOURCE	IDENTIFIER
**Chemicals, peptides, and recombinant proteins**		

Interlock fabrics	Cotton Incorporated	–

**Deposited data**		

PET and Cotton pre-reaction SEM images	Charting a path to catalytic upcycling of plastic micro/nano fiber pollution from textiles to produce carbon nanomaterials and turquoise hydrogen.	https://doi.org/10.1039/D3SU00095H

**Software and algorithms**		

Origin	OriginLab Corporation in Northampton, MA, USA.	–

**Other**		

Data repository	University of surrey open research repository	https://doi.org/10.15126/surreydata.901138

### Method details

#### Fabrics and microfibers preparation

Interlock fabrics without finishing were provided by Cotton Incorporated. The spun yarns contained 100% cotton, 100% polyester and 50%/50% blend of PET and cotton. The wet knitted interlock construction was made on a 24-cut circular knitting machine (24 needles/inch). Spun yarns from staple fibers with a size of 40/1 Ne (English Cotton Count, 40 x 840 yards of one single yarn weight 1 pound) were used to knit the fabrics. As pre-treatment, the fabrics were scoured with sodium hydroxide to remove impurities from the fibers such as wax, fats, pectin, proteins, and organic acids, and improve their wettability. Additionally, the cotton fabrics were also bleached. Micro/nanofibers or fiber fragments were produced using the Wiley Mill. The fabrics were cut with a guillotine in squares of approximately 1 cm x 1 cm. The fabric pieces were initially deconstructed in a pilot scale Wiley Mill (20 cm diameter) using a 2 mm screen. Then, the fibers produced were ground in a laboratory scale Wiley Mill (4 cm diameter) using a 40 mesh (< 420 μm) screen. The fabric pieces cannot be added directly to the laboratory scale Wiley Mill because the rotor gets stuck easily due to the thickness of the fabrics.

#### Catalyst synthesis

Bimetallic Ni-Fe catalyst with a molar ratio of 1:3 was prepared via wet impregnation method to yield a total metal loading of 10wt%, as described also in a previous study.^18^ Briefly, the necessary amounts of metal precursor (Ni (NO_3_)_2_·6H_2_O and Fe (NO_3_)_3_ 9H_2_O) were dissolved in ethanol and added to the support ϒ-Al_2_O_3_. After that, the suspensions were stirred for 4 hours at room temperature using a magnetic stirrer. The excess ethanol was removed in a rotary evaporator under reduced pressure (50°C and 150 mbar) and the resulting solid was further dried in an oven at 80°C for 12 hours. Following drying, calcination was performed at 800°C (10°C/min ramp) for 3 hours.

#### Characterization of catalysts and HTC products

##### FT-IR

Fourier-transform infrared (FTIR) spectroscopy is a non-destructive technique based on IR interaction with samples for the identification and characterization of chemical structures. It allows us to identify the variation in the functional groups during the reaction. Fourier-transform infrared (FTIR) spectroscopy measurements were conducted using a compact Alpha II FTIR spectrometer (Bruker Corporation, Billerica, MA, USA) with a diamond crystal ATR. Each powdered sample was then placed in the sample holder of the FTIR spectrometer. Spectra were recorded over the wavenumber range of 4000 to 400 cm^-1^ with a resolution of 4 cm^-1^ and an accumulation of 64 scans per spectrum. Background spectra were collected using an empty sample holder under the same conditions and subtracted from the sample spectra to remove baseline noise. Spectral data were processed using OPUS software (version 7.8, Bruker Corporation).

##### TGA-DSC

Thermogravimetric analysis (TGA) is a technique used in thermal analysis to determine the change in mass of a sample over time as the temperature varies. This measurement yields data on physical events, such as phase transitions, absorption, adsorption, and desorption. It also provides insights into chemical processes. DSC is a technique commonly used in thermal analysis in combination with the TGA analysis that provides thermograms acquired during the experiment. A thermogram is a graphical representation that shows the relationship between heat flux and either temperature or time. The thermogram analysis provides insights into the thermal transitions and thermal stability of the material. Thermogravimetric Analysis (TGA) and differential scanning calorimetry (DSC) were performed in an SDT650 apparatus from TA Instruments to measure the amount and quality of the carbon produced during HTC using temperature programmed oxidation (TPO). A total of 10 mg of sample was heated in a flow of 25 mL min^−1^ of air while the temperature was raised from room temperature to 900°C at a 5°C min^−1^ rate. The background was measured and subtracted. The instrument specifications have a dynamic temperature precision of ±0.5°C, a calorimetry Accuracy/precision of ±2% (based on metal standards), a heat capacity accuracy ±5%, a weighting accuracy of ±0.5% and a weighting precision of ±0.1%.

##### Raman spectroscopy

Raman spectroscopy is a non-destructive technique, commonly used for the characterization of carbon nanomaterials. The bands intensities, shapes and positions in the spectra provided information about the internal crystallinity of the material. Raman spectra were recorded using an InVia Reflex Raman Microscope (Renishaw, UK) with a 532 nm diode. A 50x objective was used. The Raman microscope was fitted with a cooled charged coupled detector (CCD) along with holographic notch filters and gratings tailored for each laser wavelength. The attached Leica DMLM optical microscope was equipped with different objective lenses and a trinocular viewer that accommodates a video camera, allowing direct viewing of the sample. Daily calibration of the instrument was conducted by recording the Raman spectrum of silicon in static mode. If necessary, an offset correction was performed to ensure that the position of the silicon peak to be 520 ± 1 cm ^-1^.

##### SEM-EDX

SEM is utilized to analyze the qualitatively the morphology of the samples. EDX was utilized to acquire semi-quantitative information about the elemental composition of the samples. Scanning Electron Microscopy (SEM) was carried out by using a JEOL JSM-7100F instrument, which also had an Energy Dispersive X-ray Spectroscope (EDX) analyzer for elemental analysis. Samples were prepared by mounting them on aluminum stubs using double-sided carbon adhesive tape. Prior to analysis, the samples were sputter-coated with a thin (6mm) layer of gold to enhance conductivity and reduce charging effects. SEM imaging was performed at an accelerating voltage of 2 kV(imaging) and 10 kV (EDX) with a working distance of 10 mm and 0.1 nA. Images were captured at various magnifications ranging from 100x to 10000x to obtain detailed information about the surface characteristics and structural features of the samples Carbon paint was used to fix the samples to the holder and gold coating was applied to eliminate the charging effects.

##### TEM

TEM (Transmission Electron Microscopy) was conducted using a JEOL 2100 F field emission gun electron microscope, operating at 200 kV and equipped with an Energy-Dispersive X-Ray (EDX) detector. The material was pulverized into a powder, and a small quantity was suspended in an acetone solution via an ultrasonic bath. Several drops were applied on the copper grid (Aname, Lacey carbon 200 mesh), and the solvent was allowed to evaporate at ambient temperature prior to insertion into the microscope.

##### BET

The Brunauer-Emmett-Teller (BET) equation and the Barrett–Joyner–Halenda (BJH) method were employed to determine the specific surface area and pore volume of the samples. The samples were initially degassed at 95°C under a vacuum for 4 hours. Nitrogen adsorption and desorption measurements were conducted at liquid nitrogen temperature (-195 °C) using a Micrometrics 3Flex. equipment to ascertain the textural characteristics of the catalyst and various samples.

##### HTC reaction

The HTC reactions were conducted in a 300mL batch reactor (Parr Series 5500 HPCL Reactor with a 4848 Reactor Controller) using PTFE gaskets. Six different experiments were performed: one catalytic and one non-catalytic with each feedstock (cotton, PET and a 50/50 blend of PET/cotton microfibers). In catalytic tests, 0.30 g of textile fibers, 50 g of water and 0.15 g of catalyst were loaded in a glass-lined steel vessel. In the non-catalytic test 0.30 g of textile fibers and 50 g of water were loaded in a glass-lined steel vessel. To avoid any air contamination, N_2_ was bubbled through the solution for 5 min under a stirring speed of 100 rpm before sealing the reaction vessel. Then, the reactor was heated to the reaction temperature (200°C, outogenous pressure of 22 bar), and held at this temperature for 12h under a stirring speed of 300 rpm. The pressure of the vessel was fixed according to the natural pressure generated by the solvent (water) during the reaction. After the reaction, the post-reaction sample was recovered from the liquid by centrifugation (10k rpm, 8-5 min, 1 rep) followed by drying (12 h at 80°C).

##### Pre-reaction model microfibers characterization

A fiber quality analysis was done to prove that the microfibers provided for the study were within the range of microplastics measurements. The size distribution and coarseness of the produced microfibers were measured using a HiRes Fiber Quality Analyzer (FQA) from OpTest Equipment Inc. (Ontario, Canada). For analysis, around 1 to 2 mg of microfiber samples were dispersed in 250 ml of deionized water. The FQA integrates hydraulic flow, polarized light optics, and image-processing technology to evaluate fibers suspended in water. Within the analyzer, hydrodynamic flow aligns the fibers in the detection cell, allowing measurements of fiber properties such as length, width, coarseness, percentage of fines, kink, and curl. A thorough characterization is presented in our previous research paper.^18^

### Quantification and statistical analysis

#### Enthalpy of combustion calculations

The enthalpy of oxidation for combustion events (ΔH) was calculated from the DSC thermogram with the assistance of Origin software developed by OriginLab Corporation in Northampton, MA, USA. After baseline correction, the temperature at which the transition begins (T_onset_), and the highest temperature reached during the transition (Peak temperature) were determined. The AUC (area under the curve) for the transition peak was determined by utilizing the integration tool in Origin software. The program automatically conducted all numerical integrations calculations by using the trapezoidal rule (Equation 1) and presented the findings in Joules normalized to initial weight of the sample (J/g).

Equation (1) $$AUC\;∼\;\sum_{i=1}^{n-1}\left(\frac{y_{i}+y_{i+1}}{2}\right)\Delta x$$

Where $y_{i}\;a\text{nd}\;y_{i+1}$ are heat flow values from DSC (W/g) and Δx is the difference between time intervals (s).

#### Lower heating value calculations and normalization to J/g of oxidized microfibers

To normalize the enthalpy of combustion per gram of oxidized microfibers and calculate the lower heating value for efficient comparison of all sample further calculations were necessary utilizing the data presented in Tables S1 and S2.

The lower heating value per gram of combusted sample was calculated by Equation 2 and the catalytic samples results were normalized per gram of oxidized microfiber using Equation 3.

Equation (2) $$LHV\left(J/g\right)=\frac{\Delta H_{c}}{i.w.\left(g\right)*\left(\Delta w\%\right)}$$

Where:

i.w. (g) = initial weight of sample utilized in the TPO experiment.

Δw% = The weight loss percentage from TGA data show in Table S1 for each determined exothermic thermal event.

$\Delta H_{c}=$ The enthalpy of combustion calculated using origin software are shown in Table S2 for each determined exothermic thermal event in J.

For non-catalytic samples the total microfibers is equal to the initial weight and for the catalytic products it will be the initial weight minus the weight of non-combustible catalyst (Table S1).

Equation (3) $$LHV\;notmalised\left(\frac{J}{g\;of\;microfibers}\right)=\frac{LHV\left(\frac{J}{g\;of\;sample}\right)*100}{\%w\;of\;combusted\;microfibers\;}$$

#### Total solid carbon content

The total solid carbon content of a sample was calculated as the percentage of mass combusted at the end of temperature programmed oxidation in the TGA-DSC tests (between 100°C and 900°C) from Table S1 extracted data from Figures S1 and S2. The moisture percentage (evaporated mass below 100°C), catalyst content and non-combustible material in the sample was accounted for in this calculation. Once we had the percentage of solid carbon present (Equation 4), we made this 100% of our carbon content produced and we considered as amorphous carbon those combusted below 400°C and as filamentous carbon those combusted above that temperature (Equations 5 and 6). A third consideration should be made for desorption of organic volatiles gases adsorbed in the solids; these are endothermic reactions and must be subtracted from the percentage considered as solid carbon produced. Table S1 contains all data that led to these calculations.

Equation (4) *solid carbon present= % a.c +% f.c*

% a.c = percentage of amorphous carbon from TGA data Figures S1 and S2, summarized in Table 1.

% f.c = percentage of filamentous carbon from TGA data Figures S1 and S2, summarized in Table 1.

Equation (5) $$solid\;amorphous\;carbon\;\%=\frac{\%\;a.c*100}{solid\;carbon\;present}$$

Equation (6) $$solid\;filamentous\;carbon\;\%=\frac{\%\;f.c*100}{solid\;carbon\;present}$$

For the cases where desorption of organic volatiles gasses occurs, this desorption will occur before 400°C, therefore applying the same rules the total percentage of desorbed gases (calculated from TGA data Figures S1 and S2 and summarized in Table S1) will be subtracted from the % a.c before doing the calculations of total solid amorphous carbons using Equation 5.

#### Particle size distribution

Scanning electron microscopy (SEM) images were imported into ImageJ software, where the particle size analysis was conducted manually. Setting the scale of the images and adjusting the image threshold. Individual particles were measured by selecting each particle and recording the diameter using the “Measure” function in ImageJ. The particle size measurements were imported to Origin 23 software for statistical analysis. A distribution plot was created to represent the particle size distribution, the distribution plot was configured to show particle size frequency as a function of diameter. statistical parameters, were calculated within Origin 23 to characterize the particle size distribution quantitatively.
