# Supplementary material for: Textile microfibers valorization by catalytic hydrothermal carbonization toward high-tech carbonaceous materials
Source: iScience. 2024 Nov 19;27(12):111427. doi: 10.1016/j.isci.2024.111427 (PMC11652939; doi:10.1016/j.isci.2024.111427)
Supplement: Document S1. Figures S1–S5 and Tables S1–S5 [file mmc1.pdf]

iScience, Volume *27*

Supplemental information

Textile microfibers valorization by catalytic hydrothermal carbonization toward high-tech carbonaceous materials

Silvia Parrilla-Lahoz, Marielis C. Zambrano, Joel J. Pawlak, Richard A. Venditti, Tomas Ramirez Reina, Jose Antonio Odriozola, and Melis S. Duyar

Supporting Information

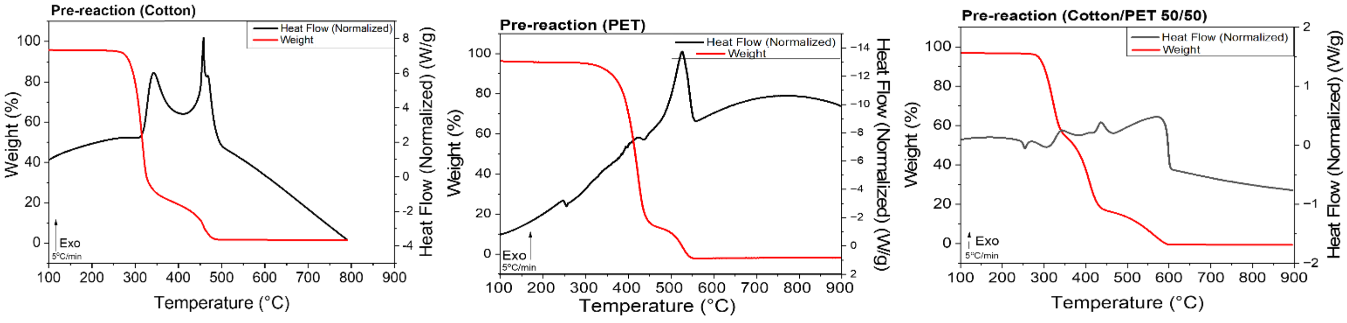

Figure 1S. TGA-DSC curves of pre-reaction samples under oxygenate atmosphere up to 900°C at 5°C/min.

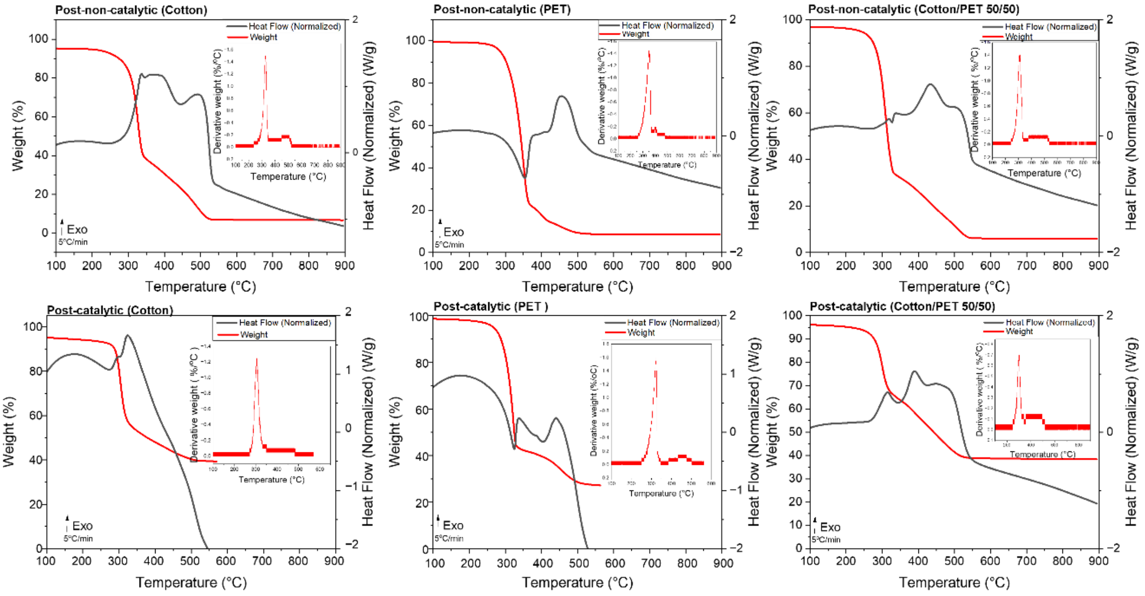

Figure 2S. TGA-DSC curves of post-reaction samples under oxygenate atmosphere up to 900°C at 5°C/min.

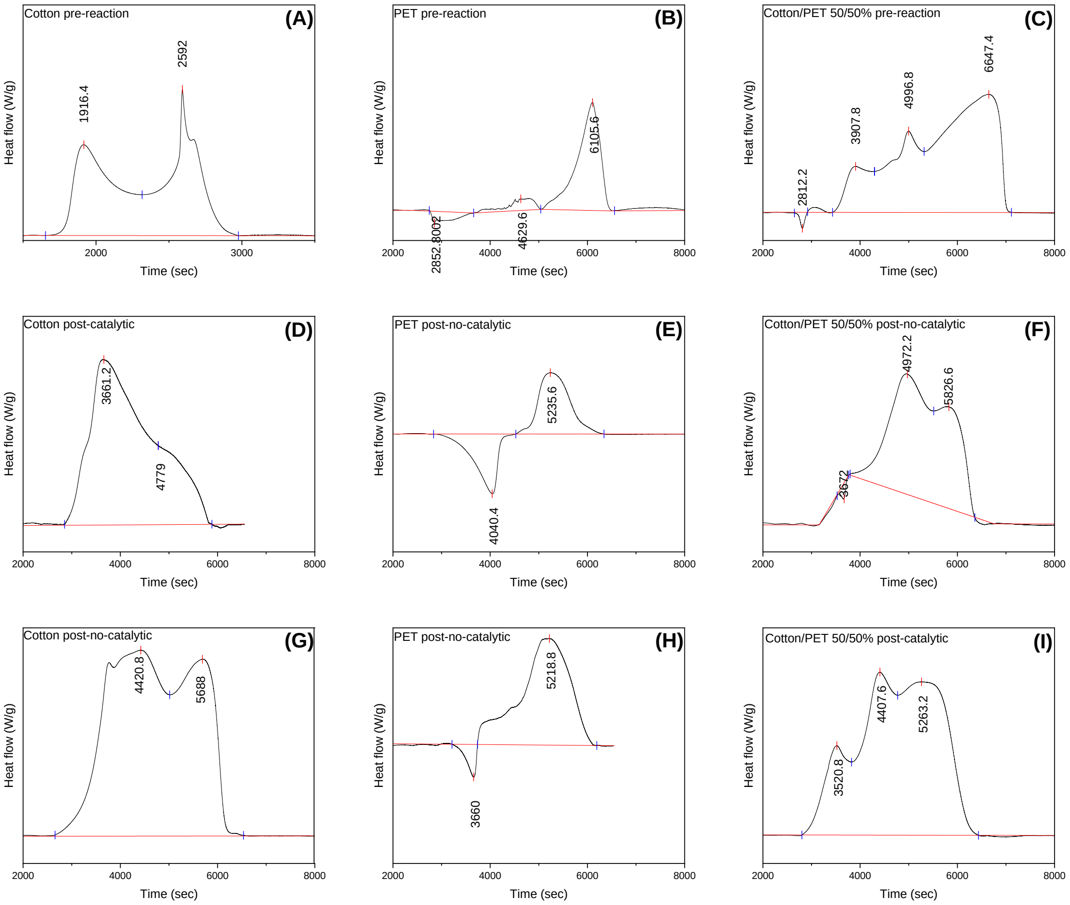

Figure 3S. TGA-DSC fitting parameters for peak integration to calculate enthalpy (J/g) data of Table 2 (Error ±5%). Related to Fig.4.

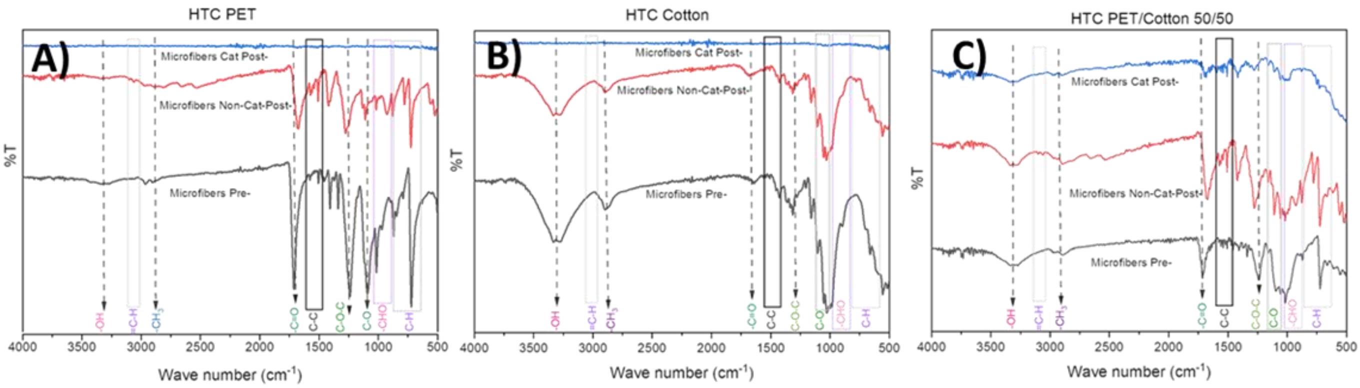

Figure 4S. Fully labelled FT-IR spectra curves for (A) PET (B) Cotton (C) PET/Cotton. Related to Fig.5.

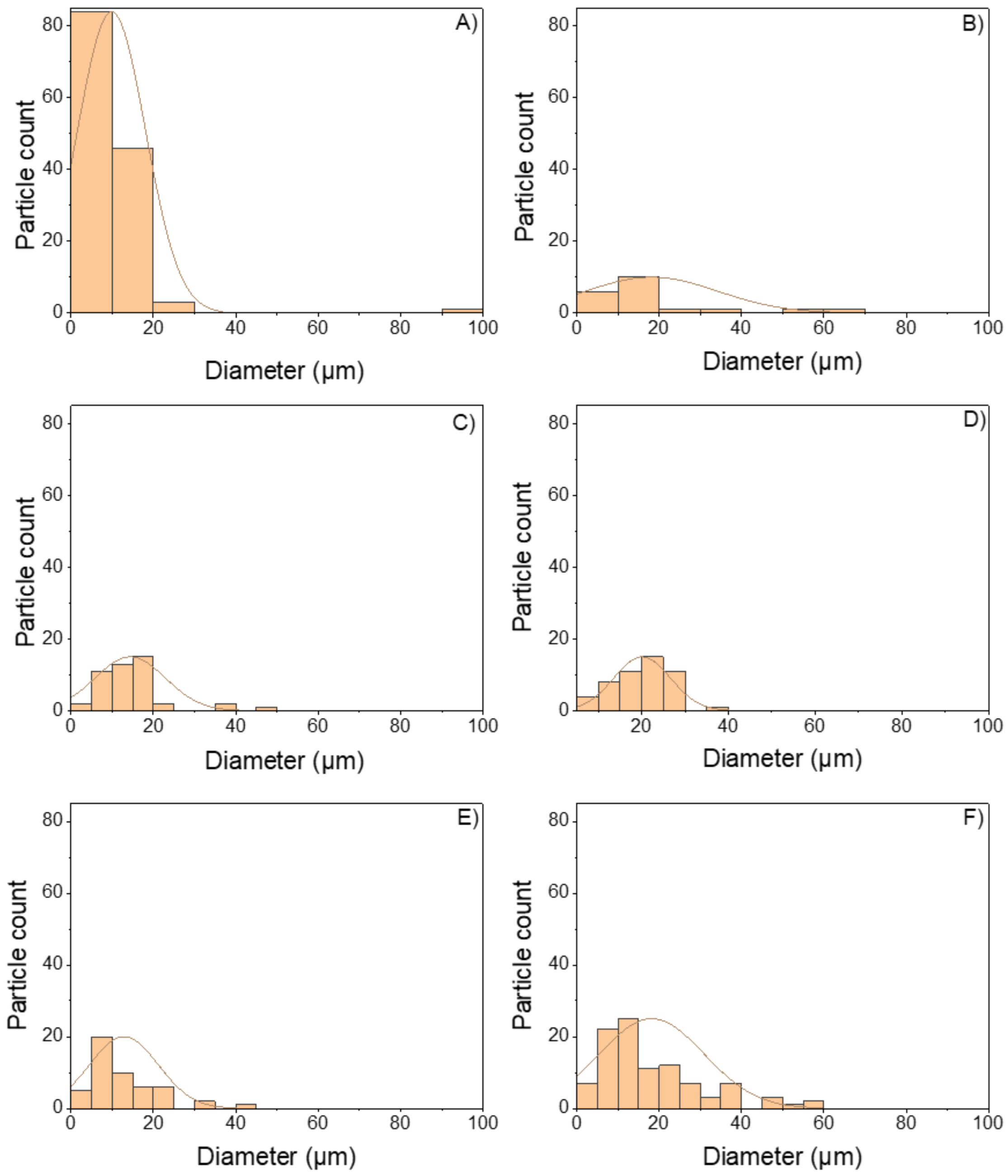

Figure 5S. Particle size distributions of (D) PET/Cotton pre-reaction physical mixture, post-non catalytic reaction samples of (A)Cotton (C)PET (E)PET/Cotton and post catalytic reaction samples of (B)Cotton (F)PET/Cotton*. Error ± 2.35. post-catalytic sample PET, have not been measured due to particle agglomeration.* Related to Fig.1.

Table 1S. TGA-DSC data. The instrument specifications have a dynamic temperature precision of ±0.5 °C, a weighting accuracy of ±0.5% and a weighting precision of ±0.1%. The error of the table calculations is ±0.5% related to weight % calculations. Therefore the max. error accumulated is ±0.5%. Related to Table 2.

Pre-reaction								
Sample	Moisture (wt.%)	Amorphous carbon (wt.%)	Amorphous C oxidation temperature (°C)	desorbed gases (wt.%)	Desorption temperature (°C)	Filamentous carbon (wt.%)	Filamentous C oxidation temperature (°C)	Non-combustibles (wt.%)
**PET**	4	82	435	0	0	14	529	0
**Cotton**	4	74	341	0	0	22	460	0
**PET/Cotton**	3	45	341	0	0	52	436-572	0
**Non-catalytic post-reaction**								
Sample	Moisture (wt.%)	Amorphous carbon (wt.%)	Amorphous C oxidation temperature (°C)	desorbed gases (wt.%)	Desorption temperature (°C)	Filamentous carbon (wt.%)	Filamentous C oxidation temperature (°C)	Non-combustibles (wt.%)
**PET**	1.5	43	350	35	350	22	409-456	0
**Cotton**	5	73	335-386	0	0	15	495	7
**PET/Cotton**	3	40	335	24	322	33	431-505	0
**FeNi catalytic post-reaction**								
Sample	Moisture (wt.%)	Amorphous carbon (wt.%)	Amorphous C oxidation temperature (°C)	desorbed gases (wt.%)	Desorption temperature (°C)	Filamentous carbon (wt.%)	Filamentous C oxidation temperature (°C)	Non-combustible catalyst (wt.%)
**PET**	3	7	337	46	324	17	441	28
**Cotton**	5	47	300-400	0	0	8	400-490	40
**PET/Cotton**	4	50	314-400	0	0	7	400-530	39

Table 2S. Combustion enthalpy change data calculated with origin software base on Figure 3S. The instrument specifications have a dynamic temperature precision of ±0.5 °C, a calorimetry Accuracy/precision of ±2% (based on metal standards), a heat capacity accuracy ±5%, a weighting accuracy of ±0.5% and a weighting precision of ±0.1%. The max. error accumulated for the calculations is ±5%. Related to Fig.4.

Sample	Peak temperature (°C)	ΔHc (J/g)	Peak temperature (°C)	ΔHc (J/g)	Peak temperature (°C)	ΔHc (J/g)	Peak temperature (°C)	ΔHc (J/g)
**Pre-reaction**								
**Cotton**	342	-1325.0	457	- 1413.4				
**PET**	255	266.6	400	-363.9	526	-2844.1		
**Cotton/PET**	255	12.5	346	-197.4	436	-428.7	575	-1112.6
**Post-no-catalytic reaction**								
**Cotton**	350	-1957.0	501	-1195.3				
**PET**	353	398.2	455	-586.5				
**Cotton/PET**	324	11.5	435	-814.3	509	-475.4		
**Post-catalytic reaction**								
**Cotton**	324	-1422.0	460	-390.3				
**PET**	322	91.3	450	-1611.6				
**Cotton/PET**	315	-374.8	390	-863.5	475	-1270.2		

Table 3S. normalize low heating values calculations data from table 1S and 2S. (LHV data have been normalize by gram of sample not taking into consideration the catalyst in J/g). The instrument specifications have a dynamic temperature precision of ±0.5 °C, a calorimetry Accuracy/precision of ±2% (based on metal standards), a heat capacity accuracy ±5%, a weighting accuracy of ±0.5% and a weighting precision of ±0.1%. Max error accumulated is ±5%.Related to Fig.4.

			amorphous phase					Filamentous phase					
**Pre-Reaction**													
sample	initial weight (mg)	initial weight (g)	thermal event temperature (°C)	weight loss (%)	ΔHc(J/g)	ΔHc(J)	LHV (J/g combusted microfiber)	thermal event temperature (°C)	weight loss (%)	ΔHc(J/g)	ΔHc(J)	LHV (J/g of combusted microfiber)	total LHV (J/g combusted microfiber)
**PET**	10.6	0.01	435.0	82.0	-363.9	-3.8	443.8	529.0	14.0	-2844.1	-30.1	20315.0	20758.8
**Cotton**	12.2	0.01	341.0	74.0	-1325.0	-16.2	1790.5	460.0	22.0	-1413.4	-17.3	6424.5	8215.1
**PET/Cotton**	25.9	0.03	341.0	45.0	-197.4	-5.1	438.7	436-572	52.0	-1541.3	-39.9	2964.0	3402.7

**Post-non- catalytic Reaction**													
sample	initial weight (mg)	initial weight (g)	thermal event temperature (°C)	weight loss (%)	ΔHc(J/g)	ΔHc(J)	LHV (J/g combusted microfiber)	thermal event temperature (°C)	weight loss (%)	ΔHc(J/g)	ΔHc(J)	LHV (J/g of combusted microfiber)	total LHV (J/g combusted microfiber)
**PET**	34.6	0.03	N	N	N	N	N	409-456	22.0	-586.5	-20.3	2665.9	2665.9
**Cotton**	20.0	0.02	335-386	73.0	-1957.0	-39.0	2680.8	495.0	15.0	-1195.3	-23.8	7968.7	10649.5
**PET/Cotton**	21.4	0.02	335.0	40.0	-814.3	-17.4	2035.8	500.0	33.0	-475.4	-10.2	1440.6	3476.4

**Post-catalytic Reaction**														
sample	initial weight (mg)	initial weight (g)	thermal event temperature (°C)	weight loss (%)	ΔHc(J/g)	ΔHc(J)	LHV (J/g combusted microfiber)	thermal event temperature (°C)	weight loss (%)	ΔHc(J/g)	ΔHc(J)	LHV (J/g of combusted microfiber)	total LHV (J/g combusted sample)	total LHV (J/g combusted microfiber)
**PET**	7.9	0.01	N	N	N		N	441.0	17.0	-1611.6	-12.8	9480.0	9480.0	13166.7

**Cotton**	4.3	0.004	300-400	47.0	- 1422.0	-6.2	3025.5	400-490	8.0	-390.3	-1.7	4878.8	7904.3	13173.8
**PET/Cotton**	27.2	0.03	314-400	50.0	-374.8	- 10.2	749.6	400-530	7.0	- 2133.7	- 58.0	30481.4	31231.0	51198.4

Table 4S. Heating values data from TGA-DSC information utilized for Figure 4. (Post-catalytic reaction products data have been normalized per gram of combusted microfiber). The instrument specifications have a dynamic temperature precision of ±0.5 °C, a calorimetry Accuracy/precision of ±2% (based on metal standards), a heat capacity accuracy ±5%, a weighting accuracy of ±0.5% and a weighting precision of ±0.1%. Max error accumulated is ±5%. Related to Fig.4.

Total heating values comparison

Sample	***Pre-reaction***	***Non-catalytic products***	***Catalytic products***
	LHV(J/g)	***LHV (J/g)***	***LHV(J/g)***
PET	20758	2665	13166
Cotton	8215	10649	13173
Cotton/PET	3402	3476	51198

Table 5S. BET measurements of pre and post reaction PET and Cotton samples.

**Sample**	**BET Surface pre-reaction (m^2/g)**	**BET surface post-reaction (m^2/g)**
**NiFe**	105	
**PET**	0.7	38
**Cotton**	0.3	55
